# Membranous and Membraneless Interfaces—Origins of Artificial Cellular Complexity

**DOI:** 10.3390/life13071594

**Published:** 2023-07-20

**Authors:** Pasquale Stano, Kanta Tsumoto

**Affiliations:** 1 Department of Biological and Environmental Sciences and Technologies, University of Salento, 73100 Lecce, Italy; 2 Division of Chemistry for Materials, Graduate School of Engineering, Mie University, 1577 Kurimamachiya-cho, Tsu 514-8507, Mie, Japan

## 1. Introduction

Living cell architecture is based on the concept of micro-compartmentation at different hierarchical levels. Cells themselves are self-bounded compartments, limited by a lipid membrane; eukaryotic cells have internal membrane-bound organelles dedicated to specific cellular functions, such as the cell nucleus, mitochondria, chloroplasts, the endoplasmic reticulum, the Golgi apparatus, endosomes, lysosomes, etc. Moreover, *membraneless* compartments are currently under intense investigation in all types of cells. Compartments imply confinement, separation, chemical gradients, interfaces, and, thus, a non-homogeneous distribution of chemical species. In other words, compartmentation is related to the generation of spatial and functional patterns, self-organization, complexity, and order (reduction in degrees of freedom by confinement). Ultimately, it can be considered a source of biological information (intended as proposed in [1,2,3]). From an even more general viewpoint, compartmentation is a pre-requisite for the fundamental property of all living systems, that is, their self-distinction from their surroundings, with the consequent onset of the system/environment duality, or—in the jargon of dynamical systems—of the statistical separation between the states of the system and the states of the environment [4]. Boundaries and the resulting compartmentation are, therefore, two related concepts which are essential for life maintenance and sustainment, as well as for regulating matter, energy, and information transfers. If we examine this from another point of view, i.e., the thermodynamic perspective, boundaries and interfaces are loci of discontinuity, and, therefore, the place where energy differences emerge are exploited (think, for example, of proton-gradient driven ATP synthesis), or where “selective” processes take places (e.g., differential permeability, adsorption, signaling, prevention of material losses).

For decades, the fascinating phenomenon of self-distinction, either via a spontaneous generation of a boundary or via separation of (micro)phases, has attracted the attention of physicists, chemists, and biologists [5] who have recognized these spontaneously occurring phenomena as driving processes for the emergence of life. Research on the systems present in nature (such as the cells and their organelles) or on systems made in the laboratory has played a decisive role in our modern understanding of these fine colloidal structures. More recently, the advent of synthetic biology (SB)—and in particular of its “bottom-up” branch—and of systems chemistry have further stimulated investigations in this field [6,7,8,9,10,11]. The goal of constructing complex cell-like systems that mimic biological systems in a stepwise and progressive manner requires the study of the compartmentation and confinement mechanisms at the root of the organization processes of living systems. That goal, however, is not merely imitative for its own sake. Instead: (i) scientists also search for the minimal complexity required to generate a target behavior; (ii) the practical and/or theoretical obstacles and the difficulties that need to be faced for the construction (or reconstruction) of structures and processes have an intrinsic value, as they reveal the physico-chemical constraints that characterize a target system and, ultimately, its structural or dynamical *organization;* and (iii) not infrequently, novel phenomena are discovered when the space of experimental parameters is systematically explored and exploited for conceptual or practical purposes. The valuable aspects of synthetic (constructive) methods which characterize SB and systems chemistry nicely complement other investigative approaches.

## 2. A Brief Description of the Collected Articles

This Special Issue was planned in order to encourage high-quality research articles in the modern framework of synthetic biology and related fields to be submitted. The starting consideration is that, to date, several biofunctions (in whole or in part) have been reconstituted in vesicles (usually, but not uniquely, made of lipids) [12,13,14], and that membraneless organelles have been recently featured as unique forms of microcompartmentation thanks to experiments with liquid/liquid phase separation (LLPS) [15,16]. Microdroplets made of hydrophilic polymer systems, similarly to the PEG/dextran aqueous two-phase systems (ATPSs), have also been used for modeling membraneless compartments [17,18,19]. Finally, various types of coacervates have shown complex cell-like behavior under several experimental conditions [20,21,22]. Through these approaches, we attempt to understand the cellular mechanisms that exploit confinement, self-bounding, and compartmentation, and that progress the field of those “artificial cell-like systems” we emphatically call “synthetic (or artificial) cells” or “protocells”. Understanding of the structure, the mechanism of their formation, and the physico-chemical dynamics of membrane-bound and membraneless compartments constitutes pre-requisite knowledge in synthetic cell research, bringing forth qualitative and quantitative developments [23,24].

This collection includes three reviews and two original reports which focus on the concepts of self-bounding and confinement, and the complex mechanisms that can be generated thereof. Shimokawa and Hamada [25] focused their review on the phenomenon of phase separation in membranes (the coexistence of liquid-ordered and liquid-disordered phases in the same membrane). This lateral separation creates compartments on the membrane’s surface, e.g., to support the localization of specific molecules for signal transduction in vivo. The review summarizes the factors that regulate phase separation, such as electrostatic interactions, chemical reactions, and membrane tension. The authors reported on the thermodynamic treatment, based on free energy and isothermal conditions, that was applied to a giant vesicle (GV) case study. The role of such knowledge on the synthetic cell perspective is highlighted (in particular, lateral phase separation can deform the membrane boundary and contribute to symmetry-breaking in very simple systems such as primitive cell models).

The theme of membrane deformation and consequent vesicle deformation is specifically explored in the next two reviews. Lagzi, Rossi, and collaborators [26] reported on the key role played by lipid membrane chemical composition in designing and constructing stimuli-responsive synthetic systems (i.e., lipid vesicles), considered as minimal cellular models, in order to investigate the division into two “daughter” vesicles so as to mimic biological cell reproduction. Several factors affect these intriguing dynamics, like solvent and ion permeability, elasticity, and response to external changes. GVs models are particularly suitable for these sorts of investigations because they allow for direct observation in real time. Physical (e.g., osmotic stress, temperature, light) as well as chemical (e.g., addition of amphiphiles and ionizable surfactants) factors are discussed, either individually or in synergy. By commenting on physico-chemical modeling and related approaches, the authors make conclusions regarding the possible usage of different strategies to control the equilibrium shapes of vesicles and on their application of combined (experimental and theoretical) investigations in the arena of synthetic cell research.

Sugawara and collaborators [27] reviewed the fascinating combination of nucleic acids, membranes, and membrane catalysts (in a model protocell) aiming at generating whole-protocell self-reproduction (in this specific case, the protocell is a DNA-containing vesicle made of special kinds of surfactants). The central point of the discussion refers to the local organization of a supramolecular catalyst which enables the membrane’s growth, followed by division (overall, the process corresponds to self-reproduction). The study includes an overview of mechanisms based on the boundaries and related reactions hosted therein. A sort of primitive “cell cycle” is also discussed. Additionally, these authors reviewed another protocell model, which was instead based on liquid/liquid phase separation (peptide droplets, i.e., a membraneless protocell model that can grow owing to “nutrients” supply—in reality, owing to activated peptide precursors—and divide by shear stress).

Sugawara, Suzuki, and collaborators also reported an original research article in which the localization of the aforementioned supramolecular catalyst was explored in more detail by means of confocal microscopy [28]. In this article, the conclusions of which are rather straightforward, it was shown that DNA co-localized with the membrane in the GV protocell model only if a cationic lipid was present, and that the actual lipo-deoxyribo-catalyst (a sort of ribozyme-like complex) was formed by dynamical exchanges. This work is the first direct observation of membrane–DNA colocalization in these model systems. Intriguingly, the authors suggest that the observed behavior resembles, at a very simple level, the mechanism of proliferation of wall-free bacteria states, known as L-forms (proliferation of the L-form does not require division machinery, and its division occurs via activation of a lipid-forming enzyme; the increased surface-area-to-volume ratio destabilizes the elongated cell, allowing it to divide).

The fifth contribution comes from Watanabe and Yanagisawa [29], who presented a detailed and quite original study on the dextran-PEG system, which generates phase separation. The novelty is the investigation of pattern formation in the dilute regime, exploiting solvent evaporation. The crucial factor that determines the course of events is the difference of the wettability and compatibility of dextran and PEG upon condensation. The unique combination of physico-chemical effects and density fluctuations plays a role in the generation of droplets, multiple domains, and strings. Intriguingly, the authors suggest that the acquired knowledge allows us to understand how the evaporation patterns of polymer blend droplets can be manipulated, changing physico-chemical parameters such as the aforementioned wettability and compatibility. The dilute regime is especially relevant in the origin of life and protocellular model investigations.

## 3. General Remarks and Conclusions

**Figure 1:**
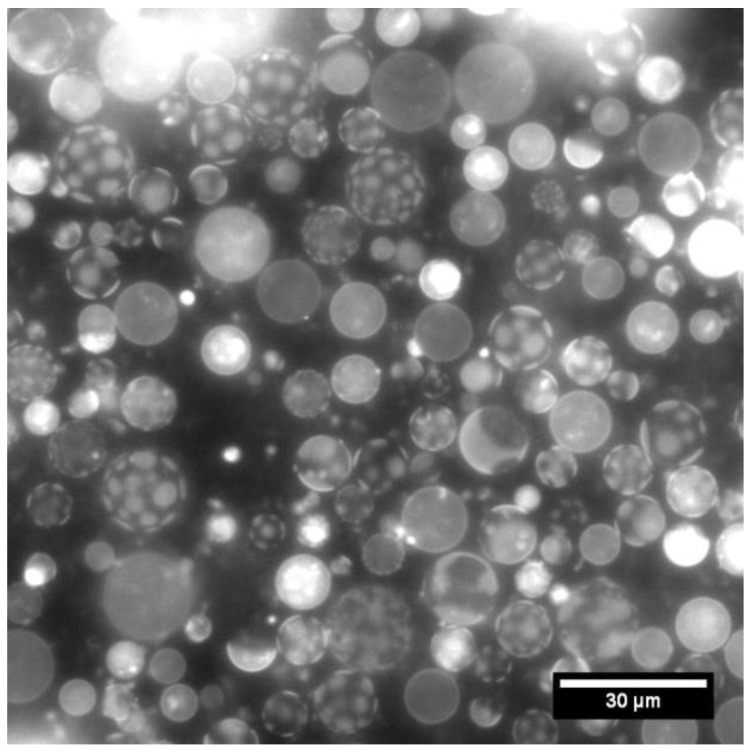
An ensemble of vesicles showing compartmentalization in two dimensions, i.e., via 2D micro-confined structures on their lipid membranes. Fluorescence microscopic images of giant vesicles with phase-separated membranes made of 1,2-dioleoyl-*sn*-glycero-3-phosphocholine (DOPC)/1,2-dipalmitoyl-*sn*-glycero-3-phosphocholine (DPPC)/cholesterol (1:2:1 in mass ratio). A small amount of rhodamine-labeled phosphoethanolamine was added as fluorescent marker. The bar represents 30 μm. Courtesy from Mr. Masahiro Mukaide (Mie University, Japan).

Several intriguing phenomena have been recently investigated by working with bottom-up synthetic cells, and most of them are related, directly or indirectly, to the peculiar features that emerge from micro-confinement and self-bounding. Others, as evidenced by the reports collected in this Special Issue, also belong to the class of phenomena that are based on the properties of boundaries and their dynamical transformations. It is interesting and important to investigate the concept of biological boundary as it is currently developed, including 2D (membranes, see Figure 1) and 3D (LLPS droplets) systems that can, potentially, also mutually interact. Vesicles, emulsion droplets, separated phases, coacervates, and similar particles are simple to design and have properties that strongly depend, often non-linearly, on chemical composition. Such an aspect implies that these systems are beautiful examples of chemical complexity, and that they show the existence of a vast chemical space to explore and, thus, the potentiality of surprising discoveries on one hand, and on the other, they reveal the challenges behind the optimization of systems to achieve pre-fixed goals. These are, in other terms, the two facets of this research; they can be purely explorative or focused to reach a predetermined pattern. Fortunately, for the latter goal, this knowledge can considerably help, guiding experimenters towards a rapid convergence of functional systems. It can be foreseen that future advancements will take advantage of this generated knowledge to prototype novel systems with higher levels of complexity. It is also fascinating that this perspective could lead to the joining of what have, until now, been considered different “alternative” compartments or boundaries (e.g., vesicle compartments and LLPS, or vesicle compartments and coacervates) to elicit novel emergent behavior in synergy. For example, boundaries could be combined and developed to create new structures [30,31].

In conclusion, we hope that the articles collected in the Special Issue “*Membranous and Membraneless Interfaces—Origins of Artificial Cellular Complexity*” will be of interest for the community of scholars working in the field at the intersection between biophysics, synthetic biology, artificial life, systems chemistry, and—in general—for all curious scientists, especially young ones. If the published studies inspire creative thoughts, consideration, novel experiments, comparisons, and critical thinking, we will consider our task to be successful and productive for the advancement of this research.
